# Changes in cardiac arrest profiles after the implementation of a Rapid Response Team

**DOI:** 10.5935/0103-507X.20210010

**Published:** 2021

**Authors:** Marina Verçoza Viana, Diego Silva Leite Nunes, Cassiano Teixeira, Silvia Regina Rios Vieira, Grazziela Torres, Janete Salles Brauner, Helena Müller, Thais Crivellaro Dutra Butelli, Marcio Manozzo Boniatti

**Affiliations:** 1 Critical Care Unit, Hospital de Clínicas de Porto Alegre, Universidade Federal do Rio Grande do Sul - Porto Alegre (RS), Brazil.; 2 Work Group of Cardiopulmonary Resuscitation, Hospital de Clínicas de Porto Alegre, Universidade Federal do Rio Grande do Sul - Porto Alegre (RS), Brazil.

**Keywords:** Heart arrest, Hospital Rapid Response Team, Parada cardíaca, Equipe de respostas rápidas de hospitais, Unidade de terapia intensiva pediástrica

## Abstract

**Objective:**

To evaluate changes in the characteristics of in-hospital cardiac arrest after the implementation of a Rapid Response Team.

**Methods:**

This was a prospective observational study of in-hospital cardiac arrest that occurred from January 2013 to December 2017. The exclusion criterion was in-hospital cardiac arrest in the intensive care unit, emergency room or operating room. The Rapid Response Team was implemented in July 2014 in the study hospital. Patients were classified into two groups: a Pre-Rapid Response Team (in-hospital cardiac arrest before Rapid Response Team implementation) and a Post-Rapid Response Team (in-hospital cardiac arrest after Rapid Response Team implementation). Patients were followed until hospital discharge or death.

**Results:**

We had a total of 308 cardiac arrests (64.6 ± 15.2 years, 60.3% men, 13.9% with initial shockable rhythm). There was a decrease from 4.2 to 2.5 in-hospital cardiac arrest/1000 admissions after implementation of the Rapid Response Team, and we had approximately 124 calls/1000 admissions. Pre-Rapid Response Team cardiac arrest was associated with more hypoxia (29.4 *versus* 14.3%; p = 0.006) and an altered respiratory rate (14.7 *versus* 4.2%; p = 0.004) compared with post-Rapid Response Team cardiac arrest. Cardiac arrest due to hypoxia was more common before Rapid Response Team implementation (61.2 *versus* 38.1%, p < 0.001). In multivariate analysis, return of spontaneous circulation was associated with shockable rhythm (OR 2.97; IC95% 1.04 - 8.43) and witnessed cardiac arrest (OR 2.52; IC95% 1.39 - 4.59) but not with Rapid Response Team implementation (OR 1.40; IC95% 0.70 - 2.81) or premonitory signs (OR 0.71; IC95% 0.39 - 1.28). In multivariate analysis, in-hospital mortality was associated with non-shockable rhythm (OR 5.34; IC95% 2.28 - 12.53) and age (OR 1.03; IC95% 1.01 - 1.05) but not with Rapid Response Team implementation (OR 0.89; IC95% 0.40 - 2.02).

**Conclusion:**

Even though Rapid Response Team implementation is associated with a reduction in in-hospital cardiac arrest, it was not associated with the mortality of in-hospital cardiac arrest victims. A significant decrease in cardiac arrests due to respiratory causes was noted after Rapid Response Team implementation.

## Abstract (pt)

**Objetivo:**

Avaliar as modificações nas características das paradas cardíacas no hospital após a implantação de um Time de Resposta Rápida.

**Métodos:**

Este foi um estudo observacional prospectivo de paradas cardíacas ocorridas no hospital entre janeiro de 2013 e dezembro de 2017. O critério de exclusão foi parada cardíaca na unidade de terapia intensiva, na emergência ou na sala cirúrgica. O Time de Resposta Rápida foi introduzido no hospital do estudo em julho de 2014. Os pacientes foram classificados em dois grupos: Pré-Time de Resposta Rápida (parada cardíaca no hospital antes da implantação do Time de Resposta Rápida) e Pós- Time de Resposta Rápida (parada cardíaca no hospital após a implantação do Time de Resposta Rápida). Os pacientes foram seguidos até a alta hospitalar ou óbito.

**Resultados:**

Ocorreram 308 paradas cardíacas (64,6 ± 15,2 anos; 60,3% homens; 13,9% com ritmo inicial chocável). Houve diminuição de 4,2 para 2,5 no índice de parada cardíaca no hospital por 1.000 admissões após o início da atuação do Time de Resposta Rápida, além de cerca de 124 chamados por 1.000 admissões. A parada antes da implantação do Time de Resposta Rápida se associou com hipóxia (29,4 *versus* 14,3%; p = 0,006) e alteração da frequência respiratória (14,7 *versus* 4,2%; p = 0,004) em comparação aos dados referentes à parada cardíaca após a implantação do Time de Resposta Rápida. Parada cardíaca por hipóxia foi mais comum antes da implantação do Time de Resposta Rápida (61,2 *versus* 38,1%; p < 0,001). Na análise multivariada, o retorno à circulação espontânea se associou com ritmo chocável (RC 2,97; IC95% 1,04 - 8,43) e parada cardíaca testemunhada (RC 2,52; IC95% 1,39 - 4,59) mas não com a implantação do Time de Resposta Rápida (RC 1,40; IC95% 0,70 - 2,81) ou sinais premonitórios (RC 0,71; IC95% 0,39 - 1,28). Na análise multivariada, a mortalidade hospitalar se associou com ritmo não chocável (RC 5,34; IC95% 2,28 - 12,53) e idade (RC 1,03; IC95% 1,01 - 1,05), porém não com a implantação do Time de Resposta Rápida (RC 0,89; IC95% 0,40 - 2,02).

**Conclusão:**

Apesar de a implantação de um Time de Resposta Rápida se associar com redução na incidência de parada cardíaca no hospital, ela não se associou com a redução da mortalidade das vítimas de parada cardíaca no hospital. Observou-se significante diminuição nas paradas cardíacas devidas a causas respiratórias após a implantação do Time de Resposta Rápida.

## INTRODUCTION

The incidence of in-hospital cardiac arrest (IHCA) is approximately 6.5 cases/1000 admissions.^(1)^^)^ Survival after IHCA is low at approximately 18%.^(1,2)^ In-hospital cardiac arrest is an often neglected condition compared with out-of-hospital cardiac arrest and other conditions.^(3)^ Based on the United Kingdom National Cardiac Arrest Audit, most patients suffering from IHCA were male (57.2), and the mean age was 73.9 years.^(4)^ The majority of IHCA patients had a non-shockable presenting rhythm (72.3%), but survival to hospital discharge was better for shockable rhythms than for non-shockable rhythms (49% *versus* 10.5%).^(4)^ The CASPRI score demonstrates that some characteristics of IHCA are associated with the outcome, such as age, initial arrest rhythm, and duration of resuscitation.^(5)^ Most IHCA is preceded by deterioration of vital signs, and the probability of survival to hospital discharge decreases with the number and severity of vital dysfunctions before arrest.^(6)^ The Rapid Response Team (RRT) assesses patients at an early stage of clinical deterioration with the aim of preventing serious adverse events in hospitalized patients. Before-and-after single-center comparison studies have shown a reduction in the rate of cardiac arrests and a greater effect with a greater “dose” of care from the RRT (i.e., a larger number of assessments per 1,000 admissions).^(7)^ However, the MERIT study, a cluster-randomized trial to evaluate the effect of RRT on the composite outcome of unexpected deaths, cardiac arrests, and unplanned intensive care unit (ICU) admissions, did not significantly reduce the incidence of these outcomes.^(8)^ Despite these findings, the Institute of Healthcare Improvement (IHI), the Joint Commission, and 2015 American Heart Guidelines for Cardiopulmonary Resuscitation and Emergency Cardiovascular recommend RRT, especially in general wards.^(9)^

However, evidence regarding whether the implementation of RRT changes the hospital survival and characteristics of IHCA (such as initial rhythm, prior deterioration of vital signs and cause of cardiac arrest) is lacking.

The aim of this study was to evaluate the impact of RRT implementation on the profile of IHCA.

## METHODS

To determine the effect of RRT implementation on the characteristics of IHCA, we conducted a cohort study using historical controls at *Hospital de Clínicas de Porto Alegre*. This is a university hospital with 800 beds, and the hospital has three ICU: a medical-surgical unit (33 beds), cardiac unit (six beds) and surgical unit (five beds). The preintervention period was between January 1^st^, 2013 and June 30, 2014, and the postintervention period was between July 1^st^, 2014 and December 31, 2017. The primary outcome is the difference in survival to hospital discharge before and after RRT implementation.

The Ethics Committee of *Hospital de Clínicas de Porto Alegre* approved the study protocol (2015-0063). Patient consent was waived because this was an observational study.

### Rapid Response Team and code team

The hospital has two different teams, the RRT, which acts when a patient presents with clinical deterioration, and the code team, which is called when the patient has cardiac arrest. An ICU senior physician leads the RRT. The general ward uses the following trigger criteria to activate RRT: need for airway management, heart rate < 40 or > 140/minutes, respiratory rate < 8/minutes or > 35/minutes, systolic blood pressure < 80mmHg or < 90mmHg with associated symptoms, a > 2-point decrease in the Glasgow Coma Scale, prolonged seizures (> 5 minutes) or laboratory triggers (pH < 7.3, bicarbonate < 12mEq/L, and lactate > 3.0mmol/L). The code team is alerted by dialing a particular number well known throughout the hospital. The call is answered in the ICU with a phone used exclusively for this purpose. The code team is composed of a senior ICU physician, a medical resident and ICU nurse, and they bring with them an emergency cart, including a manual defibrillator, airway adjuncts and supplementary medication. All hospital wards can be reached within 3 minutes.

### Definition, inclusion and exclusion criteria

Cardiac arrest was defined as when patients received chest compression, defibrillation or both.^(1)^^)^ We excluded arrests that occurred in the ICU, operating room or emergency room. Patients not admitted to the hospital at the time that cardiac arrest occurred were also excluded. All other patients who presented with cardiac arrest were included in the analysis.

### Data collection

Data were prospectively collected as part of an ongoing project to improve quality of care in the hospital. The code team completed the forms after each arrest (patient’s name, event location, initial rhythm, interventions performed), and an intensivist reviewed the data regarding vital signs prior to the arrest and hospital outcome. Patients were classified into two groups: Pre-RRT (cardiac arrest before RRT implementation) and Post-RRT (cardiac arrest after RRT implementation). Patients were followed until hospital discharge or death.

### Statistical analysis

Statistical analysis was performed using Statistical Package for Social Sciences (SPSS), version 20.0, and R, version 3.4.0 (R Foundation for Statistical Computing, Vienna, Austria). Pre-RRT and post-RRT implementation data were compared. Descriptive data are reported as the mean ± standard deviation (DP), median (interquartile range) or frequency (percentage). Nonnormally distributed variables were compared using the Mann-Whitney U test. The chi-square test was used to compare categorical variables. To examine the association between RRT implementation and survival to discharge, multivariate logistic regression analyses were used with return of spontaneous circulation (ROSC) and in-hospital mortality as the outcome variables. Covariates were included in the multivariate model if they exhibited p < 0.035 or clinical relevance.

## RESULTS

**Figure 1:**
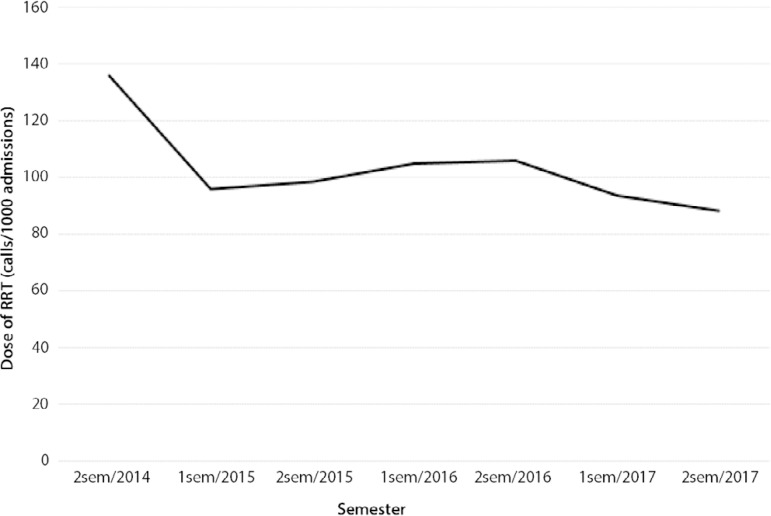
Changes over time in dose in the Rapid Response Team (call/1,000 admissions).

**Figure 2:**
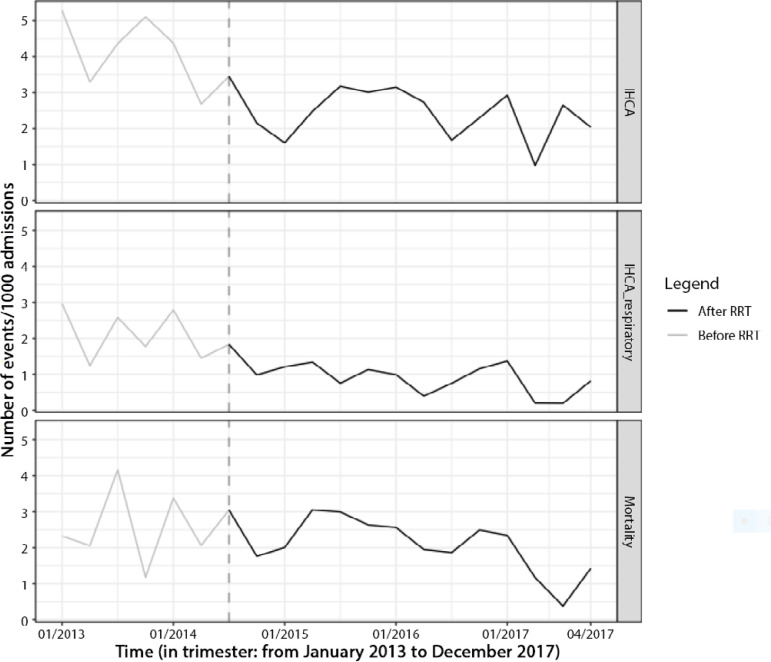
Changes over time in in-hospital mortality of cardiac arrest victims, cardiac arrest events and cardiac arrest events due to respiratory causes.

Between July 01, 2014 and December 31, 2017, the RRT was 8,956, and figure 1 shows changes in RRT dose over time (calls/1,000 admissions). Patients suffering from IHCA exhibited a mean age of 64.6 ± 15.2 years. In total, 60.3% were men. In addition, 13.9% of patients had an initial shockable rhythm, and 87% experienced in-hospital mortality. The majority of patients (61%) were included after RRT implantation due to the longer period of observation post-RRT (42 months *versus* 17 months). A reduction of 1.7 IHCA/1,000 admissions (4.2 *versus* 2.5 IHCA/1,000 admissions, p < 0.001) was noted after implementation of RRT (Figure 2). This reduction was observed mainly due to cardiac arrest of respiratory cause (2.17 to 0.98 IHCA/1,000 admissions p = 0.002) (Figure 2). The RRT had approximately 124 calls/1000 admissions. A total of 189 (60.6%) achieved ROSC and were admitted to the ICU. Of these patients, 23 (16.5%) had a limitation of treatment implemented within 24 hours of cardiac arrest. Fewer patients had hypoxia and altered respiratory rates prior to cardiac arrest after RRT implementation. Table 1 presents the main characteristics and outcomes of patients before and after RRT implementation.

RRT - Rapid Response Team.

IHCA - in-hospital cardiac arrest; RRT - Rapid Response Team.

**Table 1:** Characteristics and outcomes of patients before and after Rapid Response Team implementation

Characteristic	Pre-RRT (n = 124)	Post-RRT (n = 188)	p value
Patient characteristics			
Age (years)	64.5 ± 15.9	64.6 ± 14.8	0.963
Males	69 (53.9)	122 (64.6)	0.104
Comorbidities			
Congestive heart failure	12 (17.6)	32 (16.9)	0.893
Chronic obstructive pulmonary disease	8 (11.8)	18 (9.5)	0.599
Cancer	21 (30.9)	34 (18)	0.026
Reason for hospital admission			
Surgical	11 (16.2)	38 (20.1)	
Medical	57 (83.8)	151 (79.8)	0.242
Cardiac arrest and CPR characteristics			
Duration of hospitalization prior to cardiac arrest (days)	17 (3.75 - 37.25)	12 (5.75 - 25)	0.301
Triggers present before cardiac arrest	39 (57.4)	86 (45.5)	0.094
Heart rate < 40 or > 140/minute	3 (4.4)	13 (6.9)	0.470
Hypoxia (%)	20 (29.4)	27 (14.3)	0.006
Respiratory rate < 8 or > 30/minute	10 (14.7)	8 (4.2)	0.004
Reduction of ≥ 2 points in GCS	10 (14.7)	20 (10.6)	0.364
Main reason for cardiac arrest			
Hypovolemia	4 (3.9)	11 (6.3)	
Hypoxia	63 (61.2)	67 (38.1)	
Acidosis	9 (8.7)	12 (6.8)	
Hyperkalemia	0 (0)	6 (3.4)	
Pulmonary thromboembolism	6 (5.8)	4 (2.3)	<0.001
Acute coronary syndrome	7 (6.8)	18 (10.2)	
Others or unknown	14 (13.6)	58 (33)	
Night shift arrest	64 (52.5)	100 (52.9)	0.938
Time to arrival of code team (seconds)	100 (90 - 120)	100 (80 -120)	0.715
Witnessed cardiac arrest	85 (76.6)	94 (58.8)	0.002
Shockable rhythm	19 (15.3)	25 (13.4)	0.642
Use of adrenaline	116 (92.8)	164 (88.2)	0.182
Use of amiodarone	11 (8.9)	29 (15.5)	0.870
Outcomes			
ROSC	42 (33.9)	81 (43.1)	0.103
Limitation of treatment within 24 hours after cardiac arrest	9 (21.4)	14 (14)	0.273
Mortality	75 (87.2)	153 (86.9)	0.950

RRT - Rapid Response Team; CPR - cardiopulmonary resuscitation; GCS - Glasgow Coma Score; ROSC - return of spontaneous circulation. Results expressed as mean ± standard deviation, n (%) or median (interquartile range).

### Return to spontaneous circulation

More patients with ROSC had shockable rhythms (79.5% *versus* 57.6%, p = 0.006) and witnessed cardiac arrest (69.3% *versus* 47.8%, p < 0.001) compared with those without ROSC. No differences were between patients with and without ROSC regarding age (63.95 years ± 15.36 *versus* 65.79 ± 14.92 years; p = 0.302), premonitory signs (65.3% *versus* 54.5%; p = 0.079), night shift arrest (55.5% *versus* 44.5%; p = 0.072) or RRT implementation (56.9% *versus* 66.1%; p = 0.103). In multivariate analysis (odds ratio - OR, 95% confidence interval - 95%CI), ROSC was associated with shockable rhythm (OR = 2.97; 95%CI 1.04 - 8.43) and witnessed cardiac arrest (OR = 2.52; 95%CI 1.39 - 4.59) but not with RRT implementation (OR = 1.40; 95%CI 0.70 - 2.81) or premonitory signs (OR = 0.71; 95%CI 0.39 - 1.28).

### In-hospital mortality

Hospital mortality occurred more frequently in patients with non-shockable rhythms (90.5% *versus* 65.7%; p < 0.001) and in older patients (64.98% ± 14.95 years *versus* 58.91 ± 16.03 years; p = 0.030). No significant differences in mortality were noted regarding the presence of premonitory signs (88.7% *versus* 83.5%, p = 0.242), witnessed cardiac arrest (93.8% *versus* 85.3%; p = 0.060), night shift arrest (89.4% *versus* 84.3%; p = 0.224) or RRT implementation (86.9% *versus* 87.21%; p = 0.950) (Figure 1). In multivariate analysis, in-hospital mortality was associated with non-shockable rhythm (OR = 5.34; 95%CI 2.28 - 12.53) and age (OR = 1.03; 95%CI 1.01 - 1.05) but not with RRT implementation (OR = 0.89; 95%CI 0.40 - 2.02).

## DISCUSSION

This study evaluated the impact of RRT implementation on the characteristics and mortality of IHCA. Although we showed a strong association between the implementation of RRT and a reduction in cardiac arrest, there was no change in mortality after cardiac arrest occurred. In addition, no difference in presenting rhythm was noted before and after RRT introduction.

Early warning system scores perform well for the prediction of cardiac arrest and death within 48 hours.^(10)^ In addition, it has been shown that the survival of IHCA patients treated in general wards is lower if premonitory signs are present.^(6,11)^ The present study demonstrated that the implementation of RRT resulted in a decrease in respiratory premonitory signs in patients who suffered IHCA and consequently a reduction in cardiac arrest only due to hypoxia. However, for others with premonitory signs (hypotension, neurological disturbances), the presence of RRT was not associated with IHCA.

Up to 63% of patients with IHCA who achieve ROSC may be declared do-not-resuscitate (DNR), and 44% may have life support withdrawn.^(12)^ A RRT also has an important role in defining treatment limitations, and up to 30% of RRT calls are for end-of-life patients.^(13)^ Most of the limitations of care defined by RRT concerned patients with a diagnosis of cancer.^(14)^ We found a decrease in IHCA in patients with cancer diagnosis after RRT implementation possibly due to an increase in DNR orders. Unfortunately, data are not available to compare DNR orders before and after RRT. However, among patients with ROSC, limitation of treatment within 24 hours of arrest did not change with RRT implementation possibly due to the small sample (only 23 patients with limitation of treatment).

Our study had some strengths and limitations. First, to our knowledge, this is the only study to evaluate how RRT implementation changes the profile of IHCA. This analysis is important because it identifies areas where RRT improves the care for hospitalized patients and areas where improvement is still needed. Furthermore, it reinforces the importance of preventing IHCA given that survival is unchanged with RRT. When analyzing the causes of cardiac arrest after RRT implementation, unknown causes, hypovolemia and acute coronary syndromes were more frequent in this period compared with that before RRT. New strategies must focus on better identifying premonitory signs for these arrests and thus enabling RRT to act to prevent these events. The main limitation is the observational before-and-after design of the study in a single center. In addition, we did not have data about RRT interventions or delays. There is evidence that delay in calling RRT is associated with worst prognosis.^(15,16)^ Even in the post-RRT group, 45% of IHCA patients had triggers, suggesting a need for improvement in care to prevent cardiac arrest. Data regarding ICU bed availability were not available for this study. Reduced ICU bed availability is associated with changes in decisions on RRT and a tendency for higher rates of cardiac arrest.^(17,18)^

## CONCLUSION

In conclusion, the Rapid Response Team decreased in-hospital cardiac arrest but did not change hospital mortality after cardiac arrest occurred. It is certainly better to prevent in-hospital cardiac arrest, and a Rapid Response Team seems to be a very efficient tool for this purpose. However, even after Rapid Response Team implementation, there is still room for preventing in-hospital cardiac arrest regarding the decision to admit to the intensive care unit and interventions in patients with premonitory signs and palliative care.
